# Effect of auditory cues to lexical stress on the visual perception of gestural timing

**DOI:** 10.3758/s13414-025-03072-z

**Published:** 2025-04-30

**Authors:** Chengjia Ye, James M. McQueen, Hans Rutger Bosker

**Affiliations:** 1 Donders Institute for Brain, Cognition and Behaviour, Radboud University, Thomas Van Aquinostraat 4, 6525 GD Nijmegen, The Netherlands; 2 Max Planck Institute for Psycholinguistics, Nijmegen, The Netherlands

**Keywords:** Psycholinguistics, Speech perception, Temporal processing, Audiovisual synchrony, Beat gestures

## Abstract

Speech is often accompanied by gestures. Since beat gestures—simple nonreferential up-and-down hand movements—frequently co-occur with prosodic prominence, they can indicate stress in a word and hence influence spoken-word recognition. However, little is known about the reverse influence of auditory speech on visual perception. The current study investigated whether lexical stress has an effect on the perceived timing of hand beats. We used videos in which a disyllabic word, embedded in a carrier sentence (Experiment 1) or in isolation (Experiment 2), was coupled with an up-and-down hand beat, while varying their degrees of asynchrony. Results from Experiment 1, a novel beat timing estimation task, revealed that gestures were estimated to occur closer in time to the pitch peak in a stressed syllable than their actual timing, hence reducing the perceived temporal distance between gestures and stress by around 60%. Using a forced-choice task, Experiment 2 further demonstrated that listeners tended to perceive a gesture, falling midway between two syllables, on the syllable receiving stronger cues to stress than the other, and this auditory effect was greater when gestural timing was most ambiguous. Our findings suggest that *f*0 and intensity are the driving force behind the temporal attraction effect of stress on perceived gestural timing. This study provides new evidence for auditory influences on visual perception, supporting bidirectionality in audiovisual interaction between speech-related signals that occur in everyday face-to-face communication.

## Introduction

Speech is a multimodal phenomenon, as information is not solely encoded as soundwaves. The visual channel also plays a role, encompassing articulatory signals like the speaker’s lip movements as well as head or manual gestures, which usually co-occur with relevant speech landmarks and interact with speech (see Wagner et al., 2014, for an overview). Viewing co-speech gestures may benefit speech processing and thus facilitate communication (e.g., Holle et al., 2008; Holler et al., 2018; Nagels et al., 2015). Moreover, studies have shown that the timing of beat gestures (i.e., simple nonreferential biphasic hand movements) functions as a visual cue to the perception of speech prominence (Bosker & Peeters, 2021; Bujok et al., 2025; Treffner et al., 2008). However, little is known about the reverse: How does acoustic speech influence the visual perception of gestures? The current study examines whether auditory cues to lexical stress influence the perceived timing of beat gestures.

### Relationship between gestures and speech

While speaking, people may make gestures to facilitate communication. According to the well-accepted taxonomy developed by McNeill (1992, 2005), a co-speech manual gesture can be categorized as a deictic gesture, an iconic gesture, a metaphoric gesture, or a beat gesture. A *deictic gesture* helps to index or target a referent in a conversation through pointing. An *iconic* or *metaphoric gesture* conveys actions or affordances of a concrete referent or an abstract idea to complement the speech (e.g., by indicating the shape or size of an object). Functioning slightly differently, a *beat gesture* does not directly convey meaning but rather indicates emphasis via up-and-down hand movements.

The communicative function of gestures in audiovisual speech comprehension relies on the fact that co-speech gestures are temporally aligned with relevant speech events (e.g., Kendon, 1980; McNeill, 1992). There is evidence from naturalistic conversations. Loehr (2012) showed that the mean temporal interval between a gestural apex (i.e., the maximum extension point) and a pitch accent was 17 ms, suggesting close synchrony between gestures and speech prominence. The standard deviation, however, was 341 ms, indicating tremendous variation in temporal alignment. Gesture types also differ from each other in their alignment with speech. Iconic gestures in general co-occur with their lexical affiliates despite some variation across languages (Chui, 2005; Ferré, 2010). Centering on beat gestures, McClave (1991, 1994) revealed that downbeats co-occur with or slightly precede syllables receiving primary stress in a word and nuclei of tone units in a sentence. Corroborating evidence also comes from controlled elicited speech. Apexes of non-representational deictic, beat or even non-communicative button-pressing gestures occur within or close to the focused element in speech (Roustan & Dohen, 2010). The apex of a deictic gesture coordinates with an intonation peak; the referent thus receives joint attention (Esteve-Gibert & Prieto, 2013; Rochet-Capellan et al., 2008). In a similar vein, beat apexes usually fall within the stressed syllable and align with its *f*0 and intensity peaks (Roustan & Dohen, 2010) with the least variability, while the maximum velocity of gesture extension is concurrent with the perceptual center and vowel onset (Leonard & Cummins, 2011).

The close temporal alignment between gestures and their affiliates in speech gives rise to the question of whether and how viewing gestures influences the perception of auditory speech. To resolve this issue, one approach is to evaluate listeners’ performance in speech processing or memory tasks based on sentences or even longer discourses. Overall, listeners achieve better performance when viewing gestures congruent with their lexical affiliates in speech in both timing and meaning. Seeing gestures while listening to speech evokes faster responses to questions (Holler et al., 2018; ter Bekke et al., 2024) and makes listeners feel more addressed (Nagels et al., 2015) than only listening to speech. Listeners also give faster and more accurate responses when viewing iconic gestures that are consistent with speech in meaning than those with inconsistent meaning (Kelly et al., 2010) or meaningless hand movements (Holle et al., 2008). Although beat gestures do not convey meaning themselves, viewing these up-and-down hand movements concurrent with verbal foci may support recall of words and focused information in speech (Igualada et al., 2017; Llanes-Coromina et al., 2018; Morett & Fraundorf, 2019; So et al., 2012; Vilà-Giménez et al., 2019). The better performance is often associated with reduced cognitive load as a result of viewing gestures. However, contradictory findings under similar conditions also surface (e.g., Feyereisen, 2006). Gestures falling on unfocused words in discourse, on the contrary, may increase cognitive load and thus hinder discourse comprehension (Dimitrova et al., 2016; Morett et al., 2020; Nirme et al., 2024).

A more explicit approach is to ask what listeners hear, or more specifically, on which word in a sentence or which syllable in a word the prominence falls. The question is therefore equivalent to whether and how the perceived focus in a spoken sentence or stress in a spoken word shifts with beat timing. On the sentence level, when acoustic cues to intonation are absent, the word which is synchronous with or slightly precedes the apex is more likely to be perceived as the focused word (Treffner et al., 2008). On the word level, minimal pairs of stress are the ideal stimuli as the shift in the syllable receiving stress leads to the perception of another word; for instance, *voornaam* in Dutch means “first name” when stress is on the first syllable, but “respectable” when stress is on the second. With a series of two-alternative forced-choice (2 AFC) tasks, Bosker and Peeters (2021) exhibited that hand beats are a visual cue to lexical stress. The same acoustic recording (e.g., *voornaam*) can be perceived as having word-initial stress (*VOORnaam*, capitalization indicates the stressed syllable) when combined with a beat gesture on the first syllable, but as having word-final stress (*voorNAAM*) when the beat falls on the second syllable. The biased perception of the stress pattern thus influences spoken-word recognition. Similar results were replicated by Bujok et al. (2025), who also found that beat gestures are stronger visual cues to the perception of lexical stress than facial or lip movements. Therefore, gestural timing has a strong influence on the perception of stress in a spoken word.

A further question concerning the reverse relationship thus arises: Does lexical stress also influence the visual perception of gestural timing in return? The high variation in the temporal alignment between visual gestures and speech prominence is ubiquitous in actual conversations. Therefore, finding evidence that word stress influences the perceived timing of gestures would bear implications for how we perceive everyday multimodal signals. However, the high variation also brings challenges to answering this question. Shifting the perceived stress may lead to a definite categorical difference in spoken-word recognition as reflected by the distinct resulting words, whereas shifting the perceived beat timing is less likely to engender a comparable distinction in visual timing perception. Nonetheless, auditory influences on visual timing surface in audiovisual synchrony perception.

### Temporal ventriloquism in audiovisual synchrony perception

To decide whether an auditory event (e.g., thunder) and a visual event (e.g., lightning) are associated is challenging due to differences in (i) the speed at which sound and light are transmitted and in (ii) the time that the neural system needs to process auditory and visual signals (Alais & Carlile, 2005). Our brains cope with the asynchrony between auditory and visual signals (i.e., stimulus onset asynchrony; SOA) in three ways (Vidal, 2017). The two signals may be simply regarded as separate unimodal events so that the perceived audiovisual temporal distance is equivalent to the actual time difference between the two physical signals. Alternatively, the two signals can be temporally attracted to each other, yielding a smaller perceived audiovisual distance than the actual physical time difference. When the SOA is sufficiently small, the two signals can be completely attracted to each other and thus perceived as synchronous. In this particular case, the SOA range within which the two signals are integrated to form a fused bimodal percept defines the temporal integration window. The reduced temporal separation between auditory and visual percepts is known as *temporal ventriloquism*, in which auditory percepts have been shown to be dominant: Visual percepts are more often temporally attracted towards the auditory ones than the other way around (Bertelson & Aschersleben, 2003; Hartcher-O’Brien & Alais, 2011; Kuling et al., 2013; Vidal, 2017).

Various paradigms have been developed to measure the magnitude of attraction and to characterize the temporal integration window. The most common two are forced-choice temporal order judgements (TOJ; “Which modality is perceived first”; e.g., Alais & Carlile, 2005; Bertelson & Aschersleben, 2003; Fujisaki et al., 2004; Vroomen et al., 2004) and simultaneity judgments (SJ; “Are the two signals simultaneous or not”; e.g., Fujisaki et al., 2004; Jertberg et al., 2024; Jicol et al., 2018; van der Burg et al., 2013; van Wassenhove et al., 2007). Depending on the paradigm, the ideal temporal integration window within which simple visual and auditory signals like a light flash and a tone beep are fully attracted and perceived as synchronous ranges from ± 40 ms (Vidal, 2017; throughout this paper, a negative SOA value stands for a visual lead) to ± 100 ms (Fujisaki et al., 2004; Vroomen et al., 2004). Moreover, the SJ paradigm also revealed that the temporal integration window is wider on the visual-leading side than the visual-lagging side (Fujisaki et al., 2004; van der Burg et al., 2013), suggesting a stronger attraction effect in visual leads than in visual lags. In other words, people are more used to or more tolerant towards a slight lead in the visual stream than in the auditory stream with regard to making simultaneity judgments.

Temporal ventriloquism as well as its asymmetry are also present in higher-level speech-related bimodal events, but the case is more complicated. The canonical McGurk effect, that hearing /ba/ while seeing lip movements of /ɡa/ may lead to hearing fused /da/ (McGurk & MacDonald, 1976), typically arises within an SOA range from − 170 ms to 30 ms (van Wassenhove et al., 2007). In a recent study, Jertberg et al. (2024) confirmed this temporal window within which a fused percept prevails by asking participants what syllable they heard: /ba/, /ɡa/or /da/. Meanwhile, they also used an SJ task to formulate another temporal window within which the auditory segments and visual lip movements are perceived as synchronous. By comparing the two temporal windows as per the two tasks, Jertberg et al. (2024) demonstrated that awareness of audiovisual asynchrony does not eliminate the McGurk effect; on the contrary, the phonetic incongruence producing the fused /da/ hinders temporal attraction, making the audio and video less likely to be perceived as synchronous. Therefore, higher-level phonetic properties appear to complicate temporal processing.

One may never encounter asynchrony between lip movements and speech segments in face-to-face communication and only occasionally in videos with a delay in either the audio or video track, whereas the variable asynchrony between gestures and speech is ubiquitous. We may therefore expect similar temporal ventriloquism, and its asymmetry in synchrony perception between hand beats and prosodic prominence. To our knowledge, only Leonard and Cummins (2011) have specifically addressed synchrony perception between gestures and speech, focusing on the sentence level. Using a revised SJ task, they presented participants with two videos in each trial, both showing the same talker (face-masked) reading a sentence with one word in focus together with a co-produced beat gesture. The hand beat and the verbal focus that was naturally accented were synchronous in one video but had an SOA of ± 200 ms, ± 400 ms, ± 600 ms, or ± 800 ms in the other. Participants were asked to choose the “unnatural” one in which the auditory and visual streams were asynchronous. Results suggested that accuracy was always over 80% for gesture lags. However, accuracy for gesture leads was remarkably lower: around 33% for an SOA of − 200 ms and still lower than 60% for an SOA of − 400 ms. This asymmetry in synchrony perception between hand beats and verbal foci is consistent with findings on synchrony perception of both simple light flashes and tone beeps (Fujisaki et al., 2004; van der Burg et al., 2013) and incongruent audiovisual speech segments (Jertberg et al., 2024; van Wassenhove et al., 2007). Still, findings with these large SOA intervals cannot exhibit what happens within the SOA range from − 200 ms to 200 ms, which covers the temporal integration window between a spoken syllable and its corresponding lip movement and may also provide crucial insights into the temporal integration window between speech prominence and a hand beat. Moreover, it remains unknown how asynchrony on a word level between a beat gesture and lexical stress is processed.

### The current study

The current study aims to bridge the line of studies on temporal alignment between gestures and speech and studies on auditory-dominant temporal ventriloquism by addressing the temporal attraction between beat gestures and speech prominence. Specifically, it explores the effect of auditory cues to lexical stress on the visual perception of gestural timing. Two experiments were conducted. Disyllabic Dutch words were used as stimuli in both experiments because lexical stress in Dutch is largely reliant on three suprasegmental cues: *f*0, intensity and duration, with *f*0 being the primary cue in isolated words (Rietveld & van Heuven, 2016; Severijnen et al., 2024); unlike in English, vowels in unstressed syllables are only reduced under certain conditions in Dutch.

## Experiment 1

The first experiment examined whether the pitch peak in a stressed syllable attracts the perceived timing of a beat apex in the SOA range from − 200 ms (gesture lead) to 200 ms (gesture lag) using natural unmanipulated speech recordings. The pitch peak and the beat apex were chosen as the speech anchor point and the kinematic landmark respectively because they are coupled with the least variability in speech production (Leonard & Cummins, 2011), are easy to measure, and are easy for observers to detect. We used recordings of eight disyllabic real words in Dutch, embedded in a carrier sentence, that were naturally co-produced with a beat gesture. Half of them had stress on the first syllable, while the other half had stress on the second syllable, forming four minimal pairs of lexical stress (e.g., *VOORnaam* vs. *voorNAAM*). The variety of words and stress patterns makes it possible to generalize the attraction effect of lexical stress to other words and contexts.

A novel beat timing estimation paradigm was adopted, allowing for a measure of perceived beat timing and providing insight into the magnitude of temporal attraction. On each individual trial, participants first watched a video with a particular SOA between gesture and stress within ± 200 ms. The audio from the preceding video was then played once again. During this audio-only replay, participants were instructed to press the space bar at the moment they had perceived the beat apex in the preceding video to give their beat timing estimate. We predicted that the timing of a beat apex would be estimated to be closer to the pitch peak than it actually was. For instance, a beat apex falling 100 ms earlier than the pitch peak in the spoken word might be perceived to be only 50 ms earlier than the pitch peak, and vice versa, reflecting a reduced temporal separation between the visual landmark and the auditory anchor point (i.e., attraction). As visual leads have stronger attraction effects than visual lags in synchrony perception of speech-related stimuli (Jertberg et al., 2024; Leonard & Cummins, 2011; van Wassenhove et al., 2007), we also expected to find this asymmetry: Gesture-leading stimuli would give rise to more reduced temporal separation, and hence greater attraction than gesture-lagging stimuli.

### Methods

#### Participants

Twenty-one native listeners of Dutch (four men and 17 women, age range: 17 − 21, *M*_age_ = 19.05 years, *SD* = 1.2) participated in Experiment 1. A priori power analyses were run by means of Monte Carlo simulations (1,200 iterations; Kumle et al., 2021). To reach the power of 80%, a sample size of 16 participants was suggested for an expected effect size of 0.50 (e.g., for an SOA of 50 ms, the perceived timing of the beat apex would be shifted 25-ms closer to the pitch peak), and a sample size of 22 participants was suggested for a minimal effect size of 0.425 (85% of the abovementioned estimated effect). The R script for the power analyses is available on the online repository (see Open Practices Statement). Based on these analyses, we planned on recruiting 20 participants but received registration from 21.

All participants self-reported to have normal hearing and normal or corrected-to-normal vision and no (known) language or speech-related deficits. No detailed information about their second language(s) was collected. They were recruited from the Radboud University participants pool on a voluntary basis, and received course credits for participation. Informed consent was obtained prior to the task. The study was approved by the Ethics committee of the Faculty of Social Sciences at Radboud University (project code: ECSW-LT-2024–1–15–36,673).

#### Stimuli

In an earlier study, Cutler and van Donselaar (2001) examined spoken word recognition using 12 minimal pairs of Dutch words differing only in where the lexical stress falls. The four disyllabic pairs from their set were selected as stimuli in this study. For example, in the trochaic word *VOORnaam */ˈvoːr.naːm/ “first name,” the stress falls on the first syllable, whilst in its iambic counterpart *voorNAAM */voːr.ˈnaːm/ “respectable,” the stress falls on the second syllable. The other three minimal pairs were (1) *CAnon */ˈkaː.nɔn/ “canon” – *kaNON */kaː.ˈnɔn/ “cannon,” (2) *SERvisch */ˈsɛr.vis/ “Serbian” – *serVIES */sɛr.ˈvis/ “tableware,” and (3) *PLAto */ˈplaː.toː/ “Plato” – *plaTEAU */plaː.ˈtoː/ “plateau.”

**Fig. 1:**
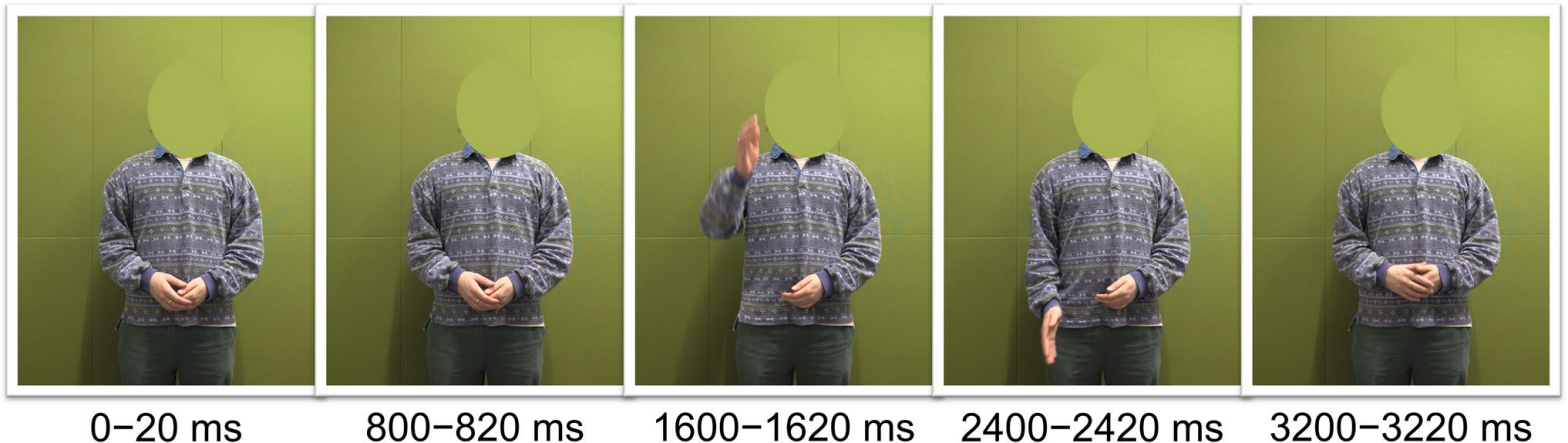
Five equally distant frames extracted from the visual stimuli. The third frame was at the end of the carrier sentence in which the speaker raised his right hand to the highest point, marking the end of the preparation phase of a beat gesture. The fourth frame was the gestural apex (the lowest point of the hand beat), functioning as the kinematic landmark in the video. In the last frame, the hand was back to the rest position

A male native speaker of Dutch was video-recorded while producing the eight words embedded in a carrier sentence: *Nu volgt het word...* (“Now follows the word...”). He was instructed to make a natural up-and-down hand beat with his dominant right hand accompanying the stress in each target word. The videos were recorded with a Canon XF405 camera with a resolution of 1,920 × 1,080 pixels at a frame rate of 50 fps, and the audios were recorded with a Shure 16 A microphone at a sampling frequency of 48 kHz. The audio recordings of all eight target words and of one carrier sentence were selected to create stimuli. More specifically, each target word was appended to a single carrier sentence of 1,290 ms that was immediately followed by an intervening silent interval of 250 ms, yielding eight auditory stimuli. We selected one video recording to be combined with all auditory stimuli—namely, that of the speaker producing *CAnon* together with the carrier sentence. This video recording lasted 3,960 ms, during which the beat apex (i.e., the lowest point of the speaker’s right hand) fell on the frame from 2,400 to 2,420 ms—hence marked as 2,410 ms (the middle of the frame). The speaker’s face was masked through postprocessing to eliminate facial articulatory cues. Figure 1 shows five equally distant frames from the video, in which the fourth exhibits the beat apex.

By aligning the beat apex in the video with the pitch peak of the stressed syllable in each audio of the eight target words using FFmpeg (FFmpeg Developers, 2023), we created eight audiovisual video clips that were labelled as having an SOA between video and audio of 0 ms. To assess the attraction effect, we further manipulated the SOAs within the range from − 200 ms (gesture leading) to 200 ms (gesture lagging) in steps of 50 ms—namely, ± 50 ms, ± 100 ms, ± 150 ms, and ± 200 ms. Note that a positive value means that the gestural apex arrived later than the pitch peak, whereas a negative value means that the gestural apex preceded the pitch peak. In total, there were 72 unique stimuli (4 minimal pairs × 2 stress patterns in each pair × 9 SOAs).

#### Procedure

Experiment 1 was run in the PsychoPy software (Version 2024.1.5; Peirce et al., 2019) in a sound-attenuating cubicle at the Donders Centre for Cognition, Radboud University. The video stimuli were presented at the center of a 24-in. full screen on a black background at a refresh rate of 120 Hz. Audio was presented through two speakers, one on each side, at a comfortable and clear volume. Participants were seated approximately 60 cm away from the screen.

Each trial involved an audiovisual video and an audio-only replay of the same speech as in the preceding video. Participants first watched the video with a particular SOA between video and audio. Then, the audio from the preceding video was played again, during which participants were asked to press the space bar at the moment they had perceived the lowest point of the speaker’s right hand. The time from the audio onset to the moment they pressed the space bar was recorded, denotated as a beat timing estimate.

At the beginning of the experiment, participants first watched two exemplar videos to become familiarized with the task, during which they were instructed to pay attention to the right-hand movement of the speaker, particularly the lowest point of his right hand. Detailed written instructions were then shown on the screen. Emphasis was placed on the fact that the timing of the lowest point of the right hand relative to the co-produced speech may vary per stimulus, so as to prevent participants from developing a strategy of simply associating the gestural apex with a single speech anchor across stimuli. The instructions were followed by 12 practice trials based on another word pair in Dutch: *VOORruit*/ˈvoːr.rœyt/“windshield”–*vooRUIT*/voː.ˈrœyt/“forward,” with SOA steps of 0 ms and ± 200 ms, each played twice (2 words × 3 SOAs × 2 repetitions).

**Fig. 2:**
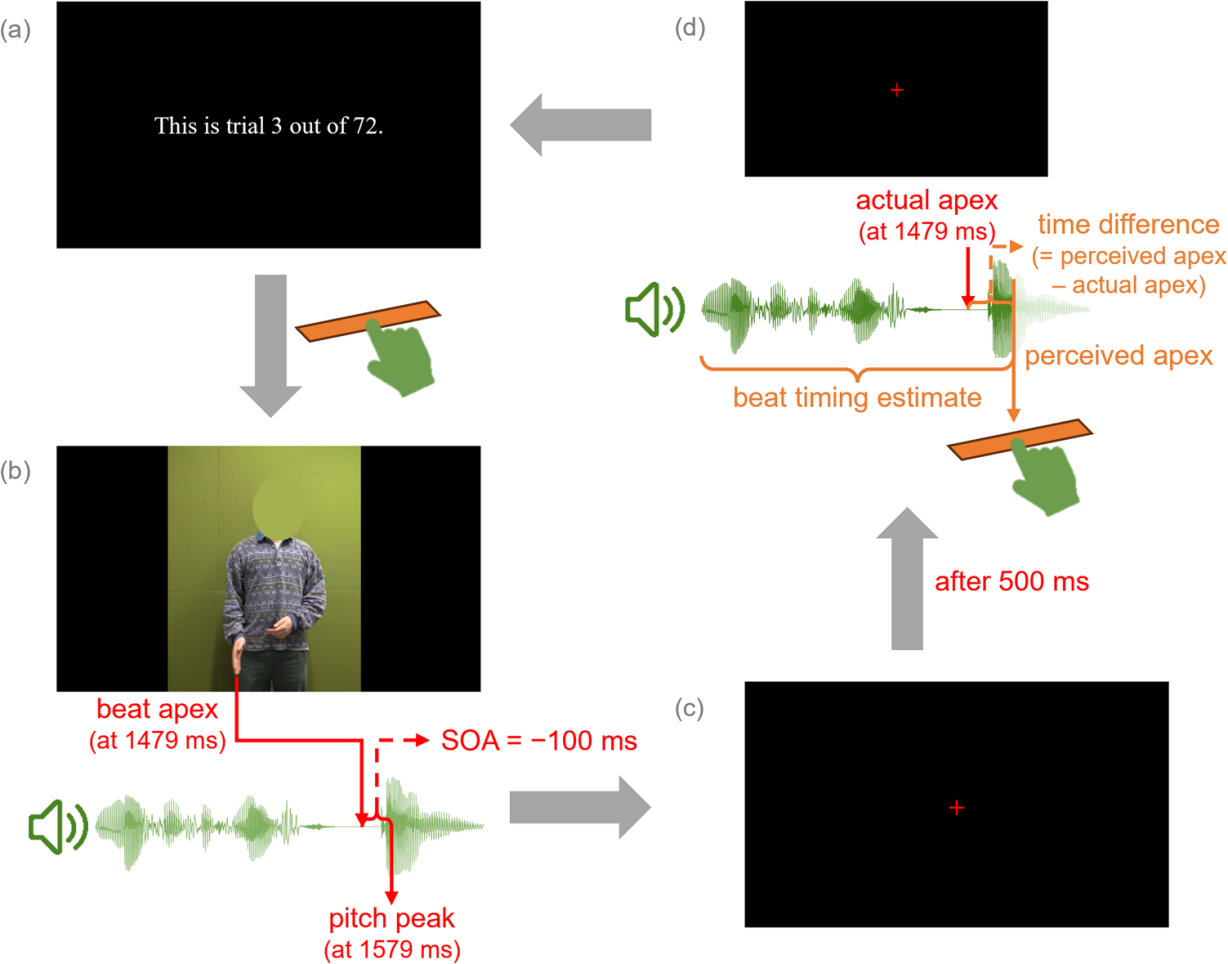
The four phases in each trial. **(a)** The *preparation* phase with trial information (written in Dutch in the experiment) that was ended by pressing the space bar. **(b)** The *video* phase, during which the audiovisual video with a particular SOA between video and audio was played once; it ended automatically after the offset of the video. **(c)** The *silence* phase that lasted 500 ms, at the beginning of which a red fixation cross was shown at the center. **(d)** The *audio replay* phase during which the audio in (b) was played again with the fixation cross remaining on the screen; participants needed to press the space bar to indicate the time they had perceived the beat apex, giving a beat timing estimate. A trial ended after the audio offset. The preparation phase of a new trial (if there was one) was then presented on the screen. (Color figure online)

During each trial, participants first saw the serial number of the upcoming trial and the total number of trials in the current session written in white at the center of a black screen. They were instructed to press the space bar every time they were ready for a new stimulus: The video then started playing. In this way, they could have complete control over the progress during the test. A red fixation cross appeared at the center of the screen right after the video offset and lasted until the end of the trial. The audio was played 500 ms after the video offset, during which participants were required to press the space bar to indicate the moment they had perceived the gestural apex. After the audio offset, the fixation cross disappeared and the trial ended. Figure 2 visualizes the process of one trial. Only during the 12 practice trials did participants receive feedback at the end of each trial about whether the moment they pressed the space bar was roughly appropriate (i.e., difference with the timing of the pitch peak was smaller than 100 ms), too fast or too slow. All instructions were written in Dutch.

No feedback was given during the real test that followed the practice. All 72 stimuli were played in a pseudorandomized order: We assigned them to eight blocks, each containing nine stimuli with nine different SOAs; the order among and within each block was fully randomized. Then there was a 5-min pause, after which the 72 stimuli were repeated in a different pseudorandomized order, yielding 144 trials per participant. The entire experiment lasted approximately 30 min.

### Results

There were altogether 3,024 beat timing estimates (21 participants × 144 trials). We calculated the *time difference* between the perceived gestural apex as reflected by the beat timing estimate and the actual gestural apex that occurred in the preceding video. For instance, the pitch peak of *CAnon* (including the preceding carrier sentence and the intervening silent interval) was at 1,579 ms; an SOA of − 100 ms meant that the beat apex fell 100 ms earlier than the pitch peak, and hence the actual timing of the apex during the video was 1,479 ms after the audio onset. If a participant pressed the space bar exactly 1,479 ms after the audio onset, perfectly replicating the actual gestural timing in the video, then the beat timing estimate would be 1,479 ms and the time difference between the perceived (1,479 ms) and actual gestural apex during the video (1,479 ms) would be 0. This would indicate no attracting effect of the pitch peak on perceived gestural timing. Instead, if a participant pressed the space bar at the time of the pitch peak, then the beat timing estimate would be 1,579 ms, hence a + 100-ms time difference as compared with the actual apex time at 1,479 ms (i.e., the apex was perceived as being 100 ms later than its actual timing). In this case, the pitch peak attracted the perceived timing of the gestural apex forward in time by 100 ms.

**Fig. 3:**
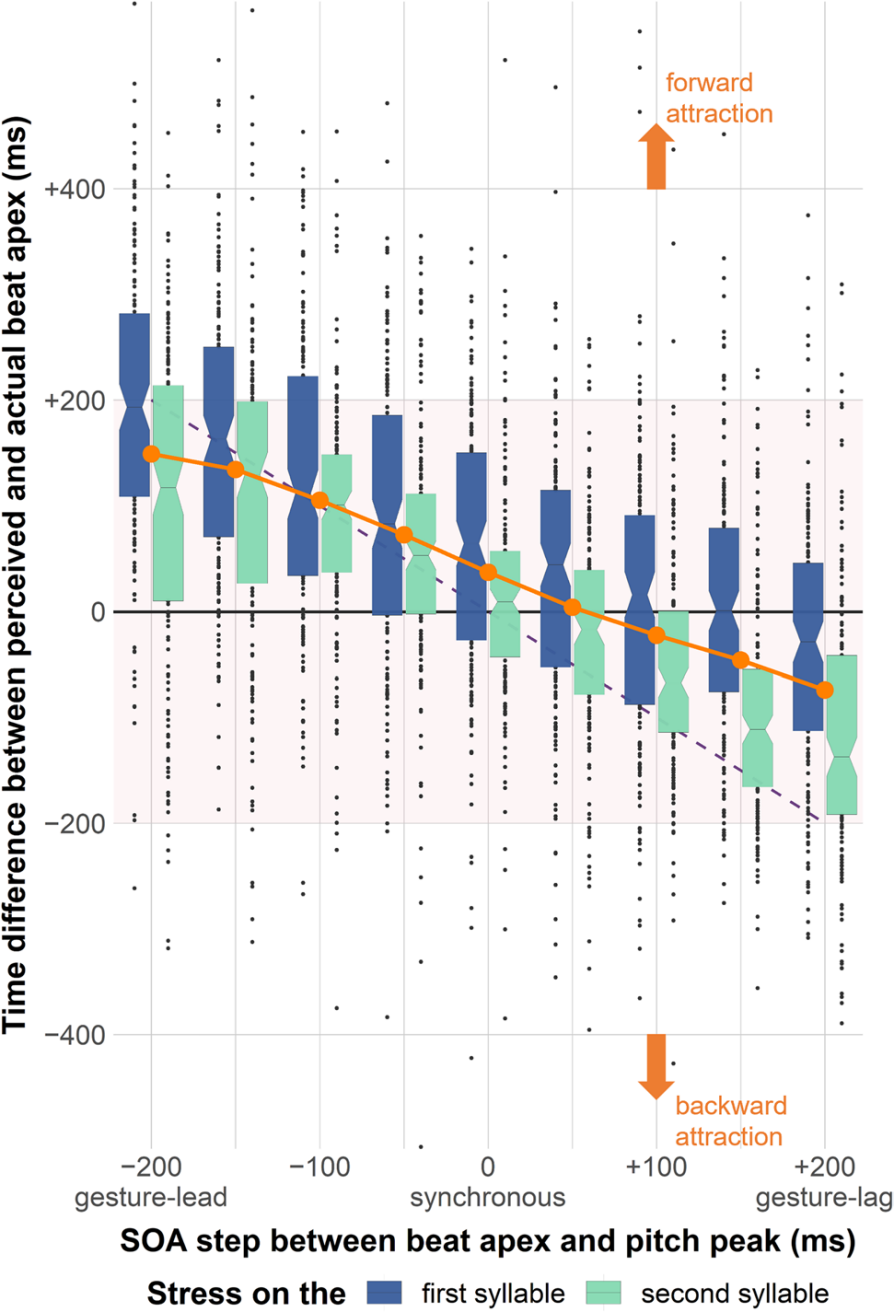
Distribution of 2,994 measurements of time difference over all SOA steps. The solid orange dots depict the means at each SOA step across the two stress patterns presented in pairs. Words with stress on the first syllable are marked in dark blue and appear on the left, while words with stress on the second syllable are marked in light green and appear on the right. Responses with *y* = 0 time difference mean that a participant pressed the space bar during the audio replay at exactly the same time as the gestural apex was presented in the preceding video, and hence, no attraction. The more distant a response is from the *y* = 0 line, the stronger the attraction effect. Responses falling above the *y* = 0 line reflect a forward attraction so that the gestural apex was perceived later than its actual timing; those below reflect a backward attraction so that the apex was perceived earlier than its actual timing. The downward slope of the orange line reflects the overall magnitude and direction of the attraction effect. The dashed purple line *y* =  *− x* in the background implies hypothetical complete attraction for reference; this illustrates space-bar presses at the time point of the acoustic pitch peak instead of the beat apex. (Color figure online)

Prior to statistical analysis of the time differences, 30 outlier responses were removed, of which 19 responses occurred before the offset of the carrier sentence (i.e., more than 250 ms before the onset of the target word); the other 11 were more than three times the standard error (3 × 198 ms) greater than the mean time difference (34 ms), thus considered too slow. The distribution of the remaining 2,994 measurements is illustrated in Fig. 3.

As the outcome *time difference* is a continuous variable, a linear mixed-effects regression (*lmer*) model was constructed in R (Version 4.4.0; R Core Team, 2024) with the *lmerTest* package (Kuznetsova et al., 2017) to assess the attraction effect. Both stress pattern and SOA step were included as fixed effects as well as their interaction. Stress pattern was a binary predictor: Words with stress on the first syllable were coded as + 0.5, and those with stress on the second syllable were coded as − 0.5. To support better convergence, SOA steps were *z*-scored (i.e., centered around the mean and divided by the *SD*). For instance, an SOA step of − 50 ms was thus converted to − 0.387. In terms of random structure, there was a random intercept per participant and by-participant random slopes for stress pattern, SOA step, and their interaction, respectively. We also included a random intercept and a random slope for stress pattern per word pair (with four levels). More complicated models failed to converge.

The model revealed a main effect of the *z*-scored SOA Step with an estimate of − 76.18. Transformed back to the original scale, the slope of the SOA step (plotted as the solid orange line in Fig. 3) had a point estimate of − 0.59 and a 95% confidence interval (95% CI) between − 0.68 and − 0.50, *t*(20.00) =  − 13.33, *p* < 0.001. In other words, the attraction effect on the perceived gestural timing was able to counteract between 50 and 68% of the SOA. Thus, for a video with an SOA step of + 100 ms in which the gestural apex is 100 ms later than the pitch peak, an average listener is likely to perceive the beat apex between 32 to 50 ms after the pitch peak. The same point estimate and a comparable 95% CI were obtained by running a model with the original values of SOA step but simplified random structure. Note that, since the 95% CI of its slope did not reach − 1, the attraction effect observed in Experiment 1 is weaker than complete attraction (as modelled with the dashed purple line *y* =  *− x* in Fig. 3). Complete attraction was not predicted and in fact would be surprising as it would reflect a situation where participants would perceive the gestural apex consistently at the time point of the acoustic pitch peak, irrespective of the actual gestural timing. Hence, this fits our expectation, showing that listeners did pay attention to the actual gestural timing in the video.

Next to this evidence for temporal attraction, the model also assessed potential evidence for *asymmetry* in the perceived synchrony between beat gestures and pitch peaks by means of its intercept. The model estimated that the intercept was not significantly greater than 0 despite its point estimate of 40 ms, *t*(8.98) = 1.99, *p* = 0.078; its 95% CI was between − 6 and 86 ms. Thus, there was no reliable evidence for stronger attraction induced by gesture leads than by gesture lags. The effect of stress pattern also turned out to be nonsignificant, *t*(3.59) = 2.06, *p* = 0.116, with a 95% CI between − 25 to 145 ms, even though it appeared in Fig. 3 that words with stress on the first syllable had a numerically greater time difference than words with stress on the second syllable. No interaction was found.

An additional exploratory one-sample *t* test was conducted to examine whether the observed time difference for the SOA step of 0 (at which the pitch peak and the gestural apex were synchronous) significantly differed from 0. Results suggested that the observed time difference here was on average 24 to 50 ms higher than 0, point estimate = 37 ms, *t*(334) = 5.57, *p* < 0.001. Therefore, listeners tended to perceive a beat apex later than a pitch peak.

### Discussion

Experiment 1 used a beat timing estimation task to investigate the synchrony perception between a hand beat and a stressed syllable. It had three main findings. The primary finding was an attraction effect of lexical stress on the perceived timing of beat gestures, as expected. The timing of a beat apex was perceived closer to the pitch peak than it actually was, at least within our SOA range of ± 200 ms. With our paradigm, the effect had a magnitude of about 50% to 68% of the SOA. Second, we did not find evidence for either an asymmetry between gesture leads and gesture lags or an effect of stress pattern. Third, we observed forward attraction at our SOA step of 0 where the beat apex and the pitch peak were synchronous. One plausible account for the last finding is that the actual point of subjective simultaneity for a beat apex and a stressed syllable occurs slightly later than the pitch peak of the stressed syllable, though the latter functioned as the auditory anchor point in Experiment 1. A comparable delay of about 17 ms for all types of gestures is reported in Loehr (2012), and that of 25 to 50 ms for beat gestures is reported in Leonard and Cummins (2011). A systematic delay or error at the moment when participants pressed the space bar may also account for the significant positive divergence from *y* = 0 (i.e., the line that indicates no attraction) at SOA = 0. However, the carrier sentence and the fixed pretarget silence of 250 ms should enable participants to relatively accurately anticipate when to press the space bar, even in trials with negative SOAs.

As the exact cause of the observed forward attraction at the synchronous point between beat apexes and pitch peaks (SOA = 0) remains unclear, we cannot draw a conclusion on asymmetry. It could be argued that the measure of attraction at each SOA step may need to be adjusted to correct for a potential systematic delay, in which case the solid orange line in Fig. 3 would need to be shifted downwards. Consequently, the actual attraction effect might be slightly weaker than reported in cases where the visual landmark leads the auditory anchor point (gesture lead) but slightly stronger in those where the visual landmark lags behind (gesture lag). However, 60% of the SOA should remain a reliable estimation of the magnitude, as the slope of the fitted line is independent from any vertical shift.

## Experiment 2

As mentioned above, although Experiment 1 demonstrated an attraction effect of lexical stress on the perceived gestural timing in general, its results are potentially susceptible to delays. In addition, as participants needed to remember the apex timing in the video with reference to auditory speech in order to be able to give their beat timing estimate later on, the beat timing estimation task involves encoding the gestural timing in memory. It may therefore be the case that the observed temporal attraction only surfaces when people retrieve the gestural timing from memory instead of being a genuine perceptual process.

To complement Experiment 1, an explicit 2 AFC task with a more controlled setting was adopted in Experiment 2, asking participants during which syllable in a disyllabic word they perceived the beat apex. The categorical decisions on whether the beat apex is perceived on the first or second syllable are the result of a perceptual process that integrates auditory syllable boundaries and visual gestural timing, and are unaffected by response delays or memory effects. Zooming in on the ambiguous SOA range between the two pitch peaks in the two syllables, we examined whether and how *f*0 and intensity as auditory cues to lexical stress bias the perception of *ambiguous* gestural timing. In other words, we were interested in how listeners determine on which syllable a beat apex falling between the two pitch peaks is perceived, given co-varying *f*0 and intensity cues.

We expected that a beat gesture timed midway between two syllables would be biased to be perceived as falling on the first syllable if *f*0 and intensity cues indicated stress on the first syllable, while the same gesture would be perceived as falling on the second syllable if *f*0 and intensity indicated stress on the second syllable. Results of Experiment 2 can thus show how *f*0 and intensity cues to lexical stress function in temporal attraction, giving insight into the role of auditory cues underlying the stress driven attraction effect on perceived gestural timing.

### Methods

#### Participants

Twenty native listeners of Dutch (seven men and 13 women, age range: 19 − 70 years, *M*_age_ = 30.85 years, *SD* = 12.9) who had not participated in Experiment 1 were recruited for Experiment 2. All were recruited on a voluntary basis following the same requirements as in Experiment 1 from the Radboud University participants pool. However, as participants in this experiment received a monetary reward instead of course credits only for students, their age range ended up being much wider than that in Experiment 1. We did not set a selecting criterion for age as we measured the responses participants made in a less time-sensitive 2 AFC task rather than the timing estimation task used in Experiment 1. As in Experiment 1, no information about the participants’ second language(s) was collected. Informed consent was obtained prior to the task. The study was approved by the Ethics committee of the Faculty of Social Sciences at Radboud University (project code: ECSW-LT-2024–4–9–33,816).

#### Stimuli

Only recordings of the isolated minimal pair *VOORnaam*–*voorNAAM* (i.e., with no carrier sentence) from Experiment 1 were used to create stimuli in Experiment 2 as both *voor* “for, before” and *naam* “name” are real words in Dutch. We could therefore transform the question from “Did the lowest point of the speaker’s right hand fall on the first syllable or the second syllable?” to “Did the lowest point of the speaker’s right hand fall on the word *voor* or *naam*?,” making it easier for naïve participants.

To control for duration, we first annotated syllable boundaries in both words using Praat (Boersma & Weenink, 2024) in order to be able to measure the duration of each syllable. We then removed duration as an auditory cue to stress. Specifically, we took the original *VOORnaam* recording, compressed the duration of the first syllable to the mean of stressed *VOOR-* and unstressed *voor-* (319 ms), and extended the duration of the second syllable to the mean of stressed *-NAAM* and unstressed *-naam* (289 ms). This procedure ensured that the temporal properties of the speech stimuli were always constant and stress-ambiguous.

**Fig. 4:**
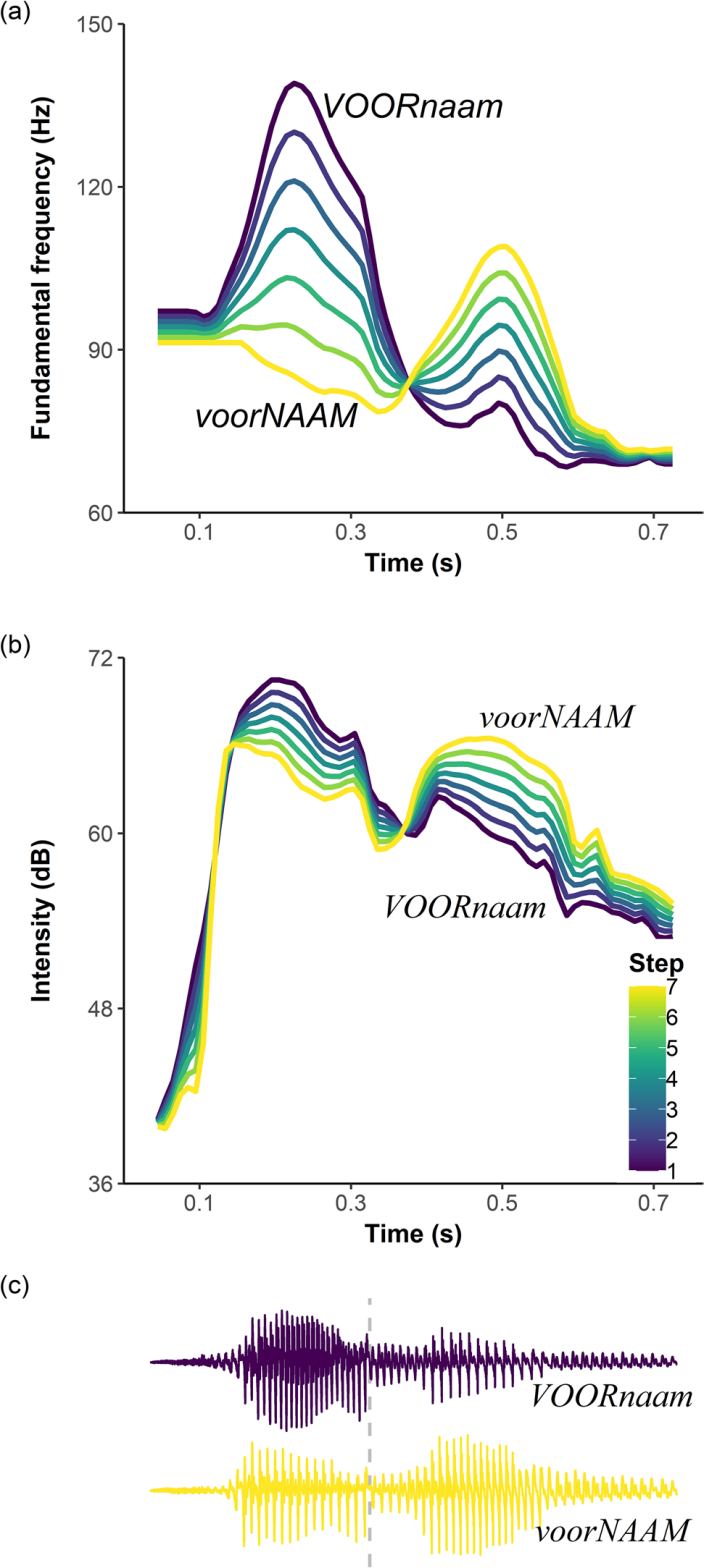
The seven steps of co-varying *f*0 (**a**; in Hz) and intensity (**b**; in dB) cues to lexical stress. Step 1 (purple) was the duration-controlled trochaic word *VOORnaam* and Step 7 (yellow) was the iambic word *voorNAAM*. Steps 2–6 were interpolated intermediate levels of equal distance. Note that Steps 2 and 6 were not used in the experiment. Panel **(c)** illustrates the oscillograms of Steps 1 and 7, with the grey dashed line *x* = 319 ms showing the syllable boundary between the two syllables. The scales of time (the *x*-axes) of all three panels are the same. (Color figure online)

We then created a Praat script to manipulate lexical stress. Five equally spaced intermediate levels of co-varying *f*0 and intensity (in time bins of 10 ms) were interpolated between the duration-controlled trochaic *VOORnaam*, to which the manipulation was applied, and iambic *voorNAAM* (see all seven levels as illustrated in Fig. 4). Step 2 (the step next to the standard *VOORnaam*) and Step 6 (the step next to the standard *voorNAAM*) were removed for their high auditory similarity to the two standard duration-controlled words with the least stress-ambiguity, thus reducing the number of auditory steps to five—namely, Steps A1, A3, A4, A5, and A7.

The video from Experiment 1 was clipped to 2,500 ms and then used as the visual stimulus in Experiment 2. The gestural apex in the clipped video fell on the frame from 1,400 to 1,420 ms. Interested in ambiguous gestural timing, we manipulated the audiovisual video so that the gestural apex in the video would fall between the pitch peak in the first syllable (230 ms) and that in the second (499 ms) in the audio using FFmpeg (FFmpeg Developers, 2023). Seven equally distant values of apex timing were interpolated between the two pitch peaks, yielding altogether nine steps of apex timing (henceforth, visual steps), with a distance of 34 ms between two adjacent steps. As for the earliest Visual Step 1 (V1), the beat apex in the video fell at 230 ms in the audio, coinciding with the pitch peak in *voor*; as for the latest Visual Step 9 (V9), the beat apex in the video fell at 499 ms in the audio, coinciding with the pitch peak of *naam*. The syllable boundary at 319 ms in the audio was between Visual Steps 3 (V3) and 4 (V4): 22 ms after V3 and 12 ms before V4. In total, there were 45 unique stimuli (5 auditory steps × 9 visual steps).

#### Procedure

The facilities and testing conditions for running Experiment 2 were the same as those for Experiment 1. Instructions written in Dutch were presented to participants prior to the test. They were informed that their task was to watch a video of a native speaker saying *VOORnaam* or *voorNAAM* while making an up-and-down movement with his right hand and then to decide on which part (*voor* or *naam*) they perceived the lowest point of the speaker’s right hand (i.e., the beat apex). If the beat apex fell on the word *voor*, which stood for the first syllable, they needed to press the left arrow on the keyboard; if it fell on *naam*, which stood for the second syllable, they needed to press the right arrow.

As in Experiment 1, there was a preparation phase at the beginning of each trial, in which the serial number of the upcoming trial and the total number of trials were presented in white at the center of a black screen. Participants needed to press the space bar to start playing the video, which allowed them to control the progress during the entire task. After the video offset, a red fixation cross was presented immediately at the center of the screen. Since that moment, participants had 3.5 s to press a key. The fixation cross disappeared after a key was pressed or after 3.5 s and then the trial ended. In the latter case, the trial received no response.

There were eight practice trials with a fixed order at the beginning of the experiment to familiarize participants with the task. The first four trials were the combination of A1 and V1, both indicating stress on the first syllable, and that of A7 and V9, both indicating stress on the second syllable; both combinations were repeated once. The last four trials were the two inconsistent combinations between audio steps and visual steps: A7 V1 and A1 V9; both were repeated once. This indicated to participants that the gestural timing and the auditory stress cues could be congruent as well as incongruent and that their responses should be informed by the visual gestural timing alone. Feedback was given immediately after each practice trial: The left key was considered correct for V1 whilst the right key was considered correct for V9.

The real test, with no feedback, followed the practice trials. Three blocks were presented, each consisting of the 45 unique stimuli in a random order, yielding 135 trials per participant. The entire experiment lasted approximately 20 min.

### Results

**Fig. 5:**
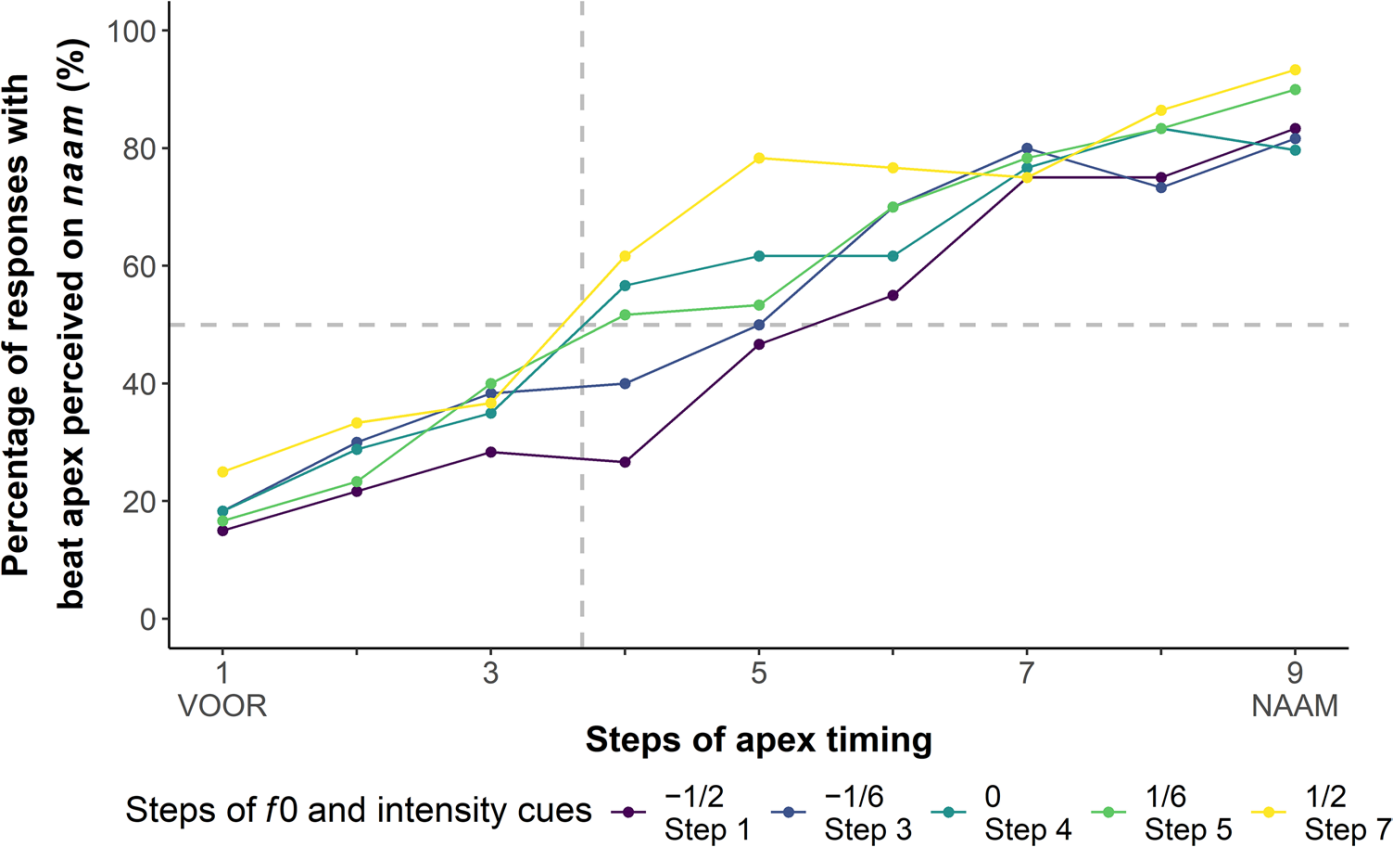
The line plot of the percentage of responses with beat apex perceived on the second syllable *naam *over nine visual steps of the timing of beat apex. Vertical differences between colored lines indicate the auditory attraction effect. All visual steps were between the pitch peaks in the two syllables of the disyllabic word *voornaam *in the audio. The distance between two adjacent steps was 34 ms. Visual Step 1 (V1), the pitch peak of *voor*, was at 230 ms in the audio and Step 9 (V9), the pitch peak of *naam*, was at 499 ms. The horizontal grey dashed line (*y *= 50) splits the plot into an upper and a lower part. Responses above this line were biased towards perceiving the beat apex on the second syllable, whereas those below were biased towards the first syllable. The vertical grey dashed line (*x *= 3.68) between Visual Steps 3 (V3) and 4 (V4) indicates the boundary between the two syllables, which was at 319 ms in the audio; it was 22 ms after V3 and 12 ms before V4. (Color figure online)

The 2,700 test trials (20 participants × 135 trials) gave rise to 2,697 dichotomous responses on the categorization of the beat apex timing (perceived on *voor* or on *naam*), as there were three trials that received no response within the required 3.5 s. Responses across all nine visual steps are illustrated in a line plot (Fig. 5). As the timing of the beat apex moved gradually from V1 that coincides with the pitch peak of *voor* to V9 on the pitch peak of *naam*, the percentage of responses with the apex perceived on *naam* gradually increased from V1 to V9, across all five auditory steps, implying an expected effect of the visual gestural timing. Most critically, the percentage of “*naam* responses” was almost always (at eight out of the nine visual steps) higher for the spoken word with the strongest *f*0 and intensity cues to stress on *naam* (A7, plotted as the yellow line in Fig. 5) than that with the strongest cues to stress on *voor* (A1, plotted as the purple line in Fig. 5). Finally, it appears that the auditory steps made a greater difference in the middle of the visual step continuum than at the two extremes.

To address these three main observations from Fig. 5, a generalized linear mixed-effects regression (*glmer*) model was constructed in order to analyze the 2,697 dichotomous responses. For the outcome, left-key responses indicating the beat apex fell on the first syllable were coded as 0, and right-key responses indicating it fell on the second syllable were coded as 1. We treated both visual steps and auditory steps as continuous variables, and scaled both of them to a range from − 0.5 to 0.5, respectively, for better intelligibility of the model results. For example, A1 of the standard duration-controlled *VOORnaam* was assigned the value − 0.5, the following A3 was transformed into − 1/6, the most ambiguous A4 was transformed into 0, and so on. As the effect of auditory cues appeared to be more pronounced in the visual steps that lay in the middle of the visual step continuum than those on the two ends, we introduced visual certainty as an extra continuous variable. This predictor ranged from 0 (the most uncertain V5, giving the least information on the categorization of beat timing for lying in the middle of the continuum) to 1 (the most certain V1 and V9, where the beat apex aligned perfectly with the pitch peak in each syllable, giving the strongest visual cue). Thus, V2 and V8 were coded as 0.75, V3 and V7 were coded as 0.5, and V4 and V6 were coded as 0.25. The largest model that could converge included these three predictors as fixed effects and two two-way interactions: one between auditory steps and visual steps, and the other between auditory steps and visual certainty. In terms of random structure, we included a random intercept and two random slopes (auditory steps and visual certainty) per participant.

This model revealed two main effects and one interaction. The first main effect was visual steps (*β* = 3.61, *SE* = 0.17, *z* = 21.58, *p* < 0.001), demonstrating that later beat apexes were more likely to be perceived to fall on the second syllable. That is, a video with V9 (with the beat apex falling on the pitch peak of *naam*) was estimated to be on average 37.07 times (the odds ratio, obtained by exponentiating the *β* coefficient) more likely to be perceived as having the apex on the second syllable than a video with V1 (i.e., with the beat apex falling on the pitch peak of *voor*)*,* the 95% CI of the odds ratio ran from 26.70 to 51.47. Critically, the second main effect was auditory steps (*β* = 1.37, *SE* = 0.28, *z* = 4.85, *p* < 0.001), successfully demonstrating stress-guided temporal attraction: The same gesture could be perceived as falling on the first syllable when combined with acoustic stress cues on *voor*, but as falling on the second syllable when stress was on *naam*. That is, a video with A7 (that of the standard *voorNAAM*, coded as 0.5 in the model) was estimated to be on average 3.92 times more likely to be perceived as having the beat apex on the second syllable than a video with A1 (that of the standard *VOORnaam*, coded as − 0.5 in the model); the 95% CI was between 2.26 and 6.80.

We also found a two-way interaction between auditory steps and visual certainty (*β* = − 0.93, *SE* = 0.45, *z* = − 2.08, *p* = 0.038). The negative coefficient indicated that the attraction effect of auditory steps was most pronounced at ambiguous visual steps (i.e., in the middle of the visual continuum) and decreased for steps with greater visual certainty. Specifically, the difference between A1 (the most *VOORnaam*-like; the purple line in Fig. 5) and A7 (the most *voorNAAM*-like; the yellow line in Fig. 5) was on average 2.54 times larger at V5 when the apex timing was the most ambiguous compared with their difference at the two extremes, V1 and V9, when the apex timing was at either of the two pitch peaks; the 95% CI was between 1.05 and 6.11. Both main effects and the two-way interaction reported here were confirmed by running an alternative model in which the difference between two adjacent auditory steps and visual steps was conventionally coded as 1, suggesting that our results were not specific to the coding scheme we used.

### Discussion

With a 2 AFC task, Experiment 2 showed that in addition to the actual gestural timing (visual steps), auditory cues to lexical stress (auditory steps, co-varying *f*0 and intensity) also make a significant difference to the visual perception of gestural timing. The categorical decision a listener makes regarding the syllable on which a beat apex falls primarily relies on the gestural timing. When the gestural apex is closer to the pitch peak in the first syllable than that in the second, a listener is more likely to perceive the apex as occurring on the first syllable, and vice versa. For the same gesture, however, its perceived timing is biased towards the syllable receiving stronger *f*0 and intensity cues to stress. Furthermore, these two suprasegmental cues under investigation have a greater effect when the gestural timing is ambiguous and thus less reliable. Combining this interaction with the nonoverlapping 95% CI of the two main effects, we may conclude that auditory cues to lexical stress serve to complement visual gestural timing cues in decisions made about which syllable received the beat apex and also that ambiguous gestural timing is more likely to be perceived as coinciding with the stressed syllable.

## General discussion

The present study investigated the effect of auditory cues to lexical stress on the visual perception of gestural timing, using two distinct but complementary tasks. The beat timing estimation task in Experiment 1 successfully exhibited stress-guided temporal attraction on the perceived timing of beat gestures within an SOA range of ± 200 ms from the pitch peak of a stressed syllable. The auditory effect reduced the perceived SOA between a beat apex and a pitch peak by around 60%. The stimuli of Experiment 1, with a variety of real words and stress patterns in a carrier sentence, are more naturalistic than the acoustically manipulated speech stimuli used in Experiment 2. To eliminate possible memory effects on the observed temporal attraction as well as to explore whether and how auditory cues to lexical stress bias the perception of *ambiguous* gestural timing, Experiment 2 adopted a canonical 2 AFC task. The more controlled setting and the manipulated co-varying *f*0 and intensity cues demonstrated that the perceived gestural timing was biased towards the syllable receiving stronger *f*0 and intensity cues to stress than the other, especially when the actual gestural timing was ambiguous. Taken together, we found evidence for a temporal attraction effect of auditory cues to lexical stress on the visual perception of gestural timing.

The temporal attraction effect between gestures and speech in our study resembles low-level temporal ventriloquism between simple light flashes and tone beeps reported in previous studies. The first similarity resides in the direction of attraction. In the low-level effect, flashes are mostly temporally shifted towards beeps, rather than the other way around (Bertelson & Aschersleben, 2003; Hartcher-O’Brien & Alais, 2011; Kuling et al., 2013; Vidal, 2017). In our study, we indeed observed that visual gestural timing was shifted towards our selected auditory anchor point (in Experiment 1) and towards the syllable receiving stronger auditory cues to stress (in Experiment 2), although this study did not address the attraction in the opposite direction (see further discussion below). Second, short exposure to audiovisual asynchrony between a flash and tone is sufficient to reduce the perceived temporal distance between the two signals, making them more likely to be perceived as synchronous. This fast-acting sensory effect also predicts the reduced temporal distance between the beat apex and the stressed syllable that was found in Experiment 1. Indeed, temporal attraction occurred immediately after exposure to the video with asynchrony between these two physical events.

However, the temporal attraction between speech-related signals also diverges from lower-level temporal ventriloquism between simple physical events. In lower-level audiovisual synchrony perception, the magnitude of the temporal ventriloquism is about 10% of the SOA (Kuling et al., 2013), much lower than the magnitude of about 60% reported in our beat timing estimation task. Note that Kuling et al.’s (2013) temporal adjustment task in rhythm perception is similar to our beat timing estimation task, in the sense that both required participants to indicate their own temporal perception rather than to make forced choices. One implication of the large discrepancy in magnitude between the two types of temporal attraction is that the extent to which people rely on the actual timing of the visual and auditory stimuli is different across the two types. In lower-level temporal ventriloquism between a light flash and tone beep, the physical signals are simple, brief, and synthesized, and there are not many cues available other than their actual timing. In contrast, both gestures and speech are continuous, unfolding over time—thus having less salient temporal anchors. Also, their temporal alignment, as well as its sizable variation (Loehr, 2012), is ubiquitous in face-to-face communication. Consequently, in higher-level temporal attraction between a beat apex and stressed syllable, previous experience with this variance may lead to greater tolerance for their asynchrony and hence less reliance on their actual timing (see Jicol et al., 2018, for an example of the influence of previous experience on synchrony perception).

Furthermore, the results of Experiment 2 indicated a stress-driven shift in the category boundary for visual gestural timing. The categorical decisions on gestural timing that participants had to make in the 2 AFC task were essentially based on syllable boundaries. Although the auditory syllable boundary was fixed, the same ambiguous beat timing was still biased towards the syllable receiving stronger *f*0 and intensity cues, as if this syllable had an expanded range. In a related vein, Baart and Vroomen (2010) revealed that speech sounds (/omso/ vs. /onso/) dubbed onto visual lip-read speech can shift the visual category boundary between the two visemes for “m” and “n,” highlighting the impact of auditory speech segments on visual categorization. However, our case is more complicated. Identifying the syllable boundary involves not only phonetic details but also phonological or even higher-level representations. As either of the two syllables alone forms a real word in Dutch (*voor* and *naam*), the process of word retrieval may also be involved. The temporal attraction therefore appears to influence higher-level cognitive processes.

As shown by Jertberg et al. (2024), phonetic properties intervene in temporal processing; in particular, incongruent auditory and visual phonetic cues hinder audiovisual synchrony perception. Our results broadened this interaction from a segmental level to a suprasegmental level. In both our tasks, a participant might use all available information to temporally locate the beat apex. Lexical stress in Dutch is primarily realized on the suprasegmental level. However, if listeners only relied on suprasegmental cues as the change in *f*0 and intensity regardless of actual asynchrony between the beat apex and stressed syllable, it is likely the case that they would simply align the perceived gestural timing to the pitch or intensity peak. This would lead to a complete attraction with a magnitude of 100% of the SOA, which conflicts with our results. Thus, the attraction was driven by *f*0 and intensity cues to stress, but was also conditioned by other factors.

One account for the incomplete attraction is that listeners attended to actual gestural timing in the visual channel as well as to the suprasegmental information. However, segmental phonetic properties work in the same direction as the visual gestural timing cue in preventing complete attraction. In Experiment 1, participants were required to indicate their perceived beat timing with reference to the spoken word, but they could use any available cues. Imagine a gesture-leading case in which the apex actually falls during the frication of /v/ at the onset of *VOORnaam*, while the pitch peak is at the following vowel /o/. Relying merely on the suprasegmental information, one may align the apex with the pitch peak within the vowel /o/. Nevertheless, the segmental information suggests the actual gestural apex co-occurred with the fricative instead of the vowel, considering the distinct acoustic properties of the two. The listener may thus remember seeing the beat apex while hearing the fricative /v/. The acoustic properties of this segment thus conflict with the *f*0 cue. As a result, a compromise on perceived beat timing needs to be reached. The beat apex is perceived to be later than its actual timing in order to align with the suprasegmental cue but constrained to be within the fricative in order to align with the segmental cue, leading hence to incomplete attraction. In Experiment 2, however, note that the segmental context at each visual timing step is exactly the same, and the only difference is the *f*0 and intensity. Under these circumstances, we still observed a large auditory-induced bias in the responses participants made, especially close to the syllable boundary where the visual beat timing cue was ambiguous. Therefore, the constraining effect of segmental cues on temporal attraction is likely limited.

To our knowledge, no theoretical model has been explicitly formulated to account for the type of temporal ventriloquism we have described. However, our findings may potentially be in line with the fuzzy logical model of perception (FLMP; Massaro, 1987), even though it has not been applied to the context of temporal processing. According to this model, multimodal speech perception follows a three-stage process. At the first stage, physical stimuli from visual and auditory sources are transformed to perceptual features that are evaluated independently in terms of prototypes generated for the specific task at hand. At the second stage, evaluated features are then integrated and matched against prototype descriptions in memory to give an overall degree of support for each prototype. A decision is made at the last stage based on the similarity between prototypes and stimulus. In the case of the temporal attraction effect on perceived beat timing, all available visual and auditory cues are compared with prototypes of beat timing with regard to lexical stress stored in memory. According to the FLMP, memory plays a crucial role, as all features are compared with the mental representations of prototypes or categories generated for the task at hand. The 2 AFC task was essentially focusing on gestural timing; this model therefore explains why visual gestural timing appears to be a stronger cue than the combination of *f*0 and intensity. In addition, this model also predicts that the contribution of one source of information to performance in the task increases as the ambiguity of the other source of information rises (Massaro & Cohen, 1993). This prediction provides a possible explanation for our findings in Experiment 2 that the auditory effect is stronger when the visual gestural timing is ambiguous. Still, note that applying the FLMP to temporal attraction effects is novel and beyond its original scope; therefore, further work on this issue is required.

The temporal attraction effect found in our study is of great relevance to everyday communication. As both gestures and speech convey linguistic information—namely, prosodic information on word stress in our case—they are prone to the lack of invariance (Liberman et al., 1967). Indeed, asynchrony between gestures and speech is ubiquitous (Loehr, 2012) and more likely to be encountered in real life, compared with asynchrony between simple artificial flashes and tones as well as that between lip movements and speech segments. Therefore, the attraction effect we demonstrate here presents an effective mechanism to cope with the vast variability in the temporal alignment between gestures and speech in human communication. Perceptually *shrinking* this variability, temporal attraction allows for a certain level of flexibility in the physical timing in both visual and auditory channels and thus benefits communication, in line with a rational noisy-channel language comprehension account (Gibson et al., 2013). Consequently, our outcomes predict that gestures that are slightly misaligned with affiliated speech may still have a comparable impact on speech perception as gestures that are synchronous with speech. We may speculate that a beat gesture that happens to precede a given word will still be as effective in cuing word-initial stress (Bosker & Peeters, 2021), in boosting recall of words or focused information in speech (Llanes-Coromina et al., 2018), and in drawing attention to the word (Dimitrova et al., 2016) as a beat gesture that is perfectly aligned to the word’s first syllable.

Although we did not examine temporal attraction in the opposite direction (i.e., visual influence on auditory events), it is plausible that beat timing may have a similar attraction effect on the perception of speech prominence. That is, the acoustic prominence in speech (e.g., a pitch peak) may also be perceived as being on a speech element closer to the moment when a gesture apex falls. This account based on temporal attraction could then explain how gestures function as visual cues to the perception of speech prominence (Bosker & Peeters, 2021; Bujok et al., 2025; Treffner et al., 2008). However, there is little evidence for this vision-driven attraction at lower perceptual levels (e.g., in the multimodal perception of simple flashes and beeps;Hartcher-O’Brien & Alais, 2011; Vidal, 2017). It thus needs to be further explored whether beat timing attracts the perceived timing of speech prominence.

There are a few more issues that need to be explored in future studies. First, our beat timing estimation task did not reveal asymmetry between gesture leads and gesture lags within the SOA range from − 200 ms to 200 ms, diverging from the finding from the SJ task in Leonard and Cummins (2011). This is likely the result of differences in experimental design. Their SJ task was more explicit and relies more on conscious synchrony discrimination than ours. Additionally, their SOA range (from − 800 ms to 800 ms) and step size (200 ms per step) are both larger than ours. Future studies using a similar timing estimation task can cover a wider SOA range to reveal at which extreme the attraction declines at a more rapid rate and hence to give more insight into the asymmetry observed by Leonard and Cummins (2011). Second, our study focused on beat gestures that do not convey meaning themselves. It thus remains unclear whether temporal attraction might also surface in the perception of deictic or iconic gestures that contain meaning themselves. Specifically, as incongruence in phonetic properties hinders temporal synchrony perception, at least on a segmental level (Jertberg et al., 2024), potential incongruency in meaning between a gesture and its lexical affiliate may also influence their temporal interaction. For instance, the temporal distance between a deictic gesture pointing to the left and its lexical affiliate in speech may be perceived as smaller when the lexical affiliate is also “left” with a congruent meaning than when it is “right” with an incongruent meaning. In other words, temporal attraction driven by a spoken word may only occur, or may be stronger, when the word shares the same meaning as the gesture.

Finally, it remains unclear whether the temporal attraction between gestures and speech observed in this study is an innate ability or an acquired behavior. Evidence for a possible innate account mainly originates from the temporal coordination (see Wagner et al., 2014, for a review) and the biomechanical link (Pouw et al., 2020) between speech and gesture production. However, it may also be argued that the temporal attraction is an acquired behavior. Native speakers of a stress language are—over the course of their lifetime—exposed to the temporal coupling between nonrepresentational gestures and word stress and its vast variability. This constant exposure may gradually shape their expectations and hence influence their temporal processing. When observing a new, perhaps slightly asynchronous pair of gesture and speech prominence, they are thus inclined to overlook the nuance of the temporal alignment. If the attraction effect is indeed built upon lifelong exposure to the temporal alignment between gestures and speech, it may vary among individuals. For example, as native speakers of a nonstress language are generally not exposed to the synchrony between gestures and word stress, they may have less or weaker temporal attraction than native speakers of a stress language. This would predict that the visual timing in one and the same audiovisual stimulus may be perceived differently depending on the perceiver’s native language. Accordingly, as iconic gestures are more likely to precede their lexical affiliates in French (Ferré, 2010) than in Mandarin Chinese (Chui, 2005), one may presume that native speakers of French perceive the same iconic gesture as occurring earlier than native speakers of Mandarin Chinese. Similarly, adults may also experience the temporal alignment differently than infants, as the amount of exposure to the temporal coupling between gestures and speech received by adults is much greater than that received by infants.

In conclusion, the present study demonstrates a temporal attraction effect of auditory cues to lexical stress on the perceived timing of a beat gesture. Experiment 1 demonstrated that its magnitude is about 60% of the SOA between the beat apex and pitch peak. Experiment 2 revealed that *f*0 and intensity give rise to this auditory effect and that it appears to influence higher-level cognitive processes, as it results in the shift of visual category boundaries of beat timing. This study provides new evidence for auditory influences on visual perception. Along with previous work showing that gestural timing influences the auditory perception of speech prominence, our results suggest that the interaction between gesture and speech in audiovisual speech perception that occurs in everyday face-to-face communication is bidirectional.

## Data Availability

The data, materials, and statistical analyses for both experiments in this study are available at 10.34973/zp61-3y60 under a CC-BY Attribution 4.0 International license. Neither of the experiments was preregistered.
